# Risk Factors Associated with Irreversible Airway Obstruction in Asthma: A Systematic Review and Meta-Analysis

**DOI:** 10.1155/2016/9868704

**Published:** 2016-03-29

**Authors:** Lanlan Zhang, Lixiu He, Jin Gong, Chuntao Liu

**Affiliations:** ^1^Department of Respiration Medicine, West China Hospital, Sichuan University, Chengdu 610041, China; ^2^Hanlin Tongjin Clinic, Chengdu 610071, China

## Abstract

Irreversible airway obstruction (IAO) is a subtype of asthma and relates to poorer prognosis in some asthma patients. However, the prevalence and risk factors for IAO are unknown. A systematic review regarding controlled clinical studies (cohort, case-control studies) on IAO asthma in adult and/or children affected by asthma/early wheeze was performed. Eighteen papers were identified in this study. It was reported that the incidence of IAO at random effects or fixed effects in severe asthma and nonsevere asthma was 0.54 (95% CI: 0.45–0.62) and 0.16 (95% CI: 0.12–0.20), respectively. In IAO asthma, the pooled odds ratio (OR) related to smoking exposure was 2.22 (95% CI: 1.82–2.73), the OR for male, smoking, and fractional exhaled nitric oxide (FENO) was 2.22 (95% CI: 1.82–2.7), 1.79 (95% CI: 1.46–2.19), and 2.16 (95% CI: 1.05–4.43), respectively, suggesting these factors increase the risk of IAO. However, a decreased OR in IAO asthma was observed due to rhinitis (OR = 0.31, 95% CI: 0.24–0.40), atopy (OR = 0.584, 95% CI: 0.466–0.732), and atopic dermatitis (OR = 0.60, 95% CI: 0.42–0.85), indicating these factors are associated with reduced risk of IAO. IAO in asthma is associated with gender, smoking, FENO, rhinitis, atopy, and atopic dermatitis.

## 1. Introduction

Asthma is a multifactorial, heterogeneous, and chronic inflammatory disease, which is characterized with the symptoms of cough, shortness of breath, wheeze, and chest tightness. The majority of patients with reversible airflow limitation asthma can be easily controlled by regular medication. However, in a small subset of asthmatic patients, even aggressive treatment fails to control the disease. The prevalence of these patients with so-called irreversible airway obstruction (IAO) asthma is not known.

Backman et al. first reported the patients with IAO asthma [1]. Lange et al. [2] found that asthmatic patients had greater declines in forced expiratory volume in 1 second (FEV1) over time compared to those who did not have asthma. Ulrik and Backer [3] found that IAO adults patients usually are associated with moderate to severe asthma. Some risk factors for IAO development have been defined; IAO asthma is associated with more severe disease [4–6] and is a predictor of overall mortality in asthmatic patients [7]. The reduction of risk factors for IAO may be the key strategy to decrease the morbidity in severe asthmatic patients with IAO. Vonk et al. [8] found that 41% of asthmatic patients did not have airway obstruction and 16% of asthmatic patients had IAO. However, studies about the prevalence and risk factors for IAO in asthmatic patients are limited and often contradictory. Martin et al. demonstrated that males have higher possibility than females to develop into asthma patients [9]. Ten Brinke et al. [6] found that the onset of asthma in adults increased AHR and sputum eosinophilia, which increased the risk factor for IAO asthma. Apostol et al. [10] found that smoking is a risk factor for IAO asthma. Aspirin sensitivity has been identified as a risk factor for IAO asthma [11].

Our meta-analysis was designed to assess the prevalence and risk factors of IAO in a well-defined group of patients with asthma/nonsevere asthma and to examine the clinical characteristics. In this study, we analyzed age, gender, disease duration, rhinitis, atopic dermatitis, aspirin sensitivity, smoking history and atopic status, and some clinical characteristics including markers of airway inflammation, fractional exhaled nitric oxide (FENO), and percentage of eosinophils and neutrophils in induced sputum.

## 2. Methods

According to the rules of the meta-analysis of Observational Studies in Epidemiology group, this meta-analysis was conducted [12]. The literature related to the terms “airways obstruction” OR “irreversible airway obstruction” OR “fixed airflow obstruction” OR “airway remodeling” AND “asthma” was collected from Ovid Medline, Pubmed, and ISI database by two independent reviewers (Z. LL. and G. J., last update in March, 2013). English was included in this meta-analysis. References were also manually searched to identify additional published or unpublished data and were evaluated by experts.

Studies of our meta-analysis included the following inclusion criteria: (1) primary outcome of incidence or risk factors of IAO asthma; (2) follow-up of at least 70% of patients; (3) all studies that used self-reported, physician-diagnosed asthma or new symptoms and/or medication use compatible with asthma as the criteria for incident asthma diagnosis. We excluded the studies that used single asthma symptom as an outcome and did not establish asthma diagnosis. Moreover, we did not consider those studies that did not report the odds risk. Study data sources were examined to ensure that every included dataset was unique.

Stata 12.0 (Stata Corporation, College Station, TX) [13] was used to calculate ORs using inverse-variance weighted, random-effects meta-analysis [14, 15]. Random-effects methodology was chosen to analyze within-study and between-study variations. Heterogeneity of data was evaluated using the *Q*-statistic [15]. We weighted the study-specific adjusted log odd risks for cohort studies and the study-specific adjusted log odds ratios for case-control studies by inversing their variance to compute a pooled odds risk and 95% confidence intervals (CIs) on a forest plot [16], and publication bias was evaluated [16, 17]. Using data from studies that provided mean repeat length of specific case groups, we calculated standardized mean difference (SMD) to compare IAO cases with reversible airway obstruction (RAO) controls. The SMD and 95% CI were calculated in each group of cases. A plan was established prior to performing sensitivity analyses for identified issues relative to study quality, if necessary, rather than applying weights to studies in the meta-analysis based simply on quality scoring criteria [12].

*χ* ^2^-based *Q* statistic evaluated heterogeneity and considered the statistical significance at *I* ^2^-value less than 50%. When the *I* ^2^-value is less than 50%, fixed-effect model was used; Otherwise, a random-effects model was applied. The significance of the pooled OR was determined by the *Z*-test, and *P* less than 0.05 was considered statistically significant. Publication bias was analyzed by several methods: (i) funnel plots; (ii) Egger's test that was also used to statistically assess publication bias. All statistical tests were performed by using the Revman 5.0 software and Stata 12.0.

## 3. Results

According to our search terms, 343 references were retrieved, but only 32 articles met our inclusion criteria. Among them, some references that only presented cross-sectional data and failed to present any measurement of the odds risk for IAO were further excluded [2, 9, 13, 18–32]. Only 18 studies were included in this study [3–6, 8, 11, 30–40], which included one study from persistent wheezing but not from asthma [41]. All 18 cohort studies were published between 1995 and 2013 (Table 1).

## 4. Incidence of IAO Asthma

**Figure 1:**
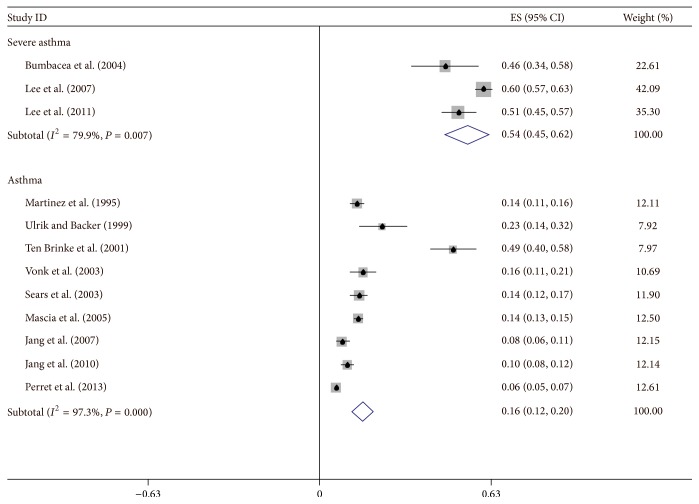
The summary for the odds of incident IAO asthma by a random-effects model. Numbers indicate reference number.

Twelve articles with the data of risk factors for IAO were retrieved [3–6, 8, 11, 33, 34, 36, 38, 39, 41]. Of the total 13,498 patients, 16% of patients were diagnosed with IAO. Four articles were involved in severe asthma [4–6, 34], in which 54% of patients were analyzed by asthma subtypes and IAO asthma using the random-effects model. The incidence of IAO asthma in non-severe asthma patients was 16% with the random-effects model (Figure 1) or 8% with the fixed-effect model.

## 5. Risk Factors

### 5.1. Male

**Figure 2:**
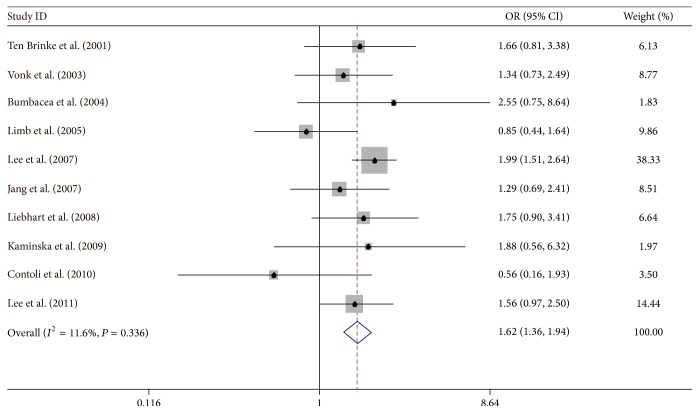
Meta-analysis for the associations between IAO asthma risk and male gender.

Ten studies [4, 6, 8, 31, 32, 34–37, 42] were carried out in a case-control study, the pooled odds risk for male by fixed-effect model was 1.622 (95% CI: 1.358–1.937), and there was a considerable heterogeneity among studies (*I* ^2^ = 11.6%) (Figure 2).

### 5.2. Smoking

**Figure 3:**
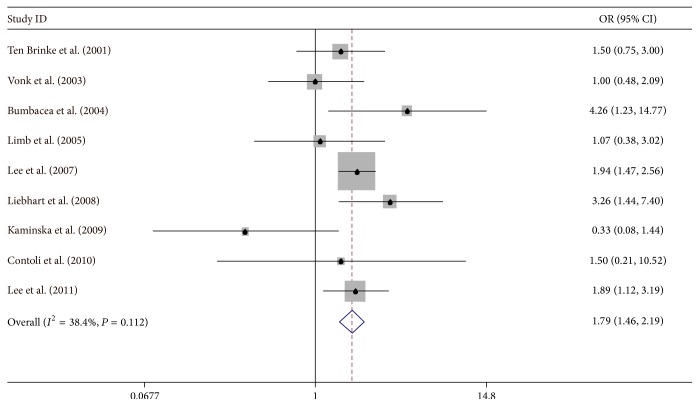
Meta-analysis for the associations between IAO asthma risk and smoking.

Eight articles were retrieved based on smoking for IAO asthma patients in a cohort study [4–6, 33–35, 37, 39]. The amount of heterogeneity was decreased (*I* ^2^ = 38.2%) when we set our analysis to case-control studies; the patients exposed to smoking showed the pooled OR of 1.79 (95% CI: 1.46–2.19). Moreover, we did not find any evidence of publication bias (*P* value of Egger's symmetry test: 0.606) (Figure 3).

### 5.3. Atopy

**Figure 4:**
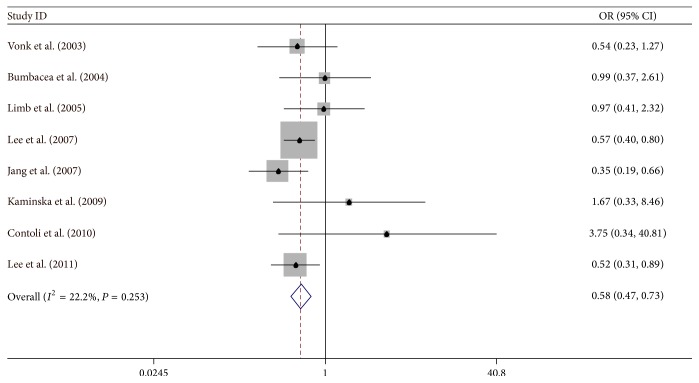
Meta-analysis for the associations between IAO asthma risk and rhinitis.

Eight studies related to atopy were included in this study [4, 5, 8, 31, 32, 34–36]; the overall OR was 0.584 (95% CI: 0.466–0.732) by fixed-effect model. There was a small heterogeneity *I* ^2^ = 22.2% (Figure 4).

### 5.4. Rhinitis

**Figure 5:**
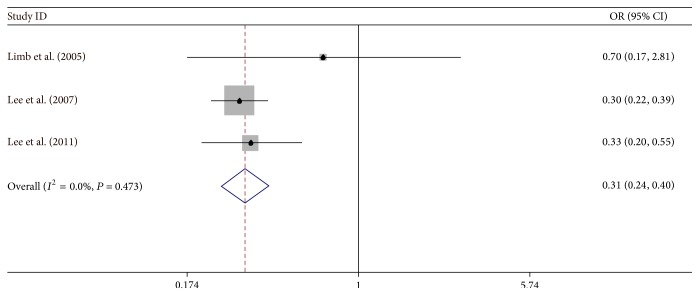
Meta-analysis for the associations between IAO asthma risk and atopy.

Three studies were associated with rhinitis [5, 35, 41]. Rhinitis showed a protective effect on IAO asthma (OR = 0.31, 95% CI: 0.24−0.40) by a fixed-effect model. There was no heterogeneity (*I* ^2^ = 0%) between IAO asthma studies and RAO asthma studies (Figure 5).

### 5.5. Aspirin Sensitivity

Two studies relative to aspirin sensitivity were included [5, 11]. The fixed-effect model generated the OR of 2.053 (95% CI: 1.417–2.689), but there was significant heterogeneity by random-effects model (*I* ^2^ = 94.4%). Although the result showed the heterogeneity, both of them showed that aspirin sensitivity was an important risk factor for IAO.

### 5.6. FENO

Two studies related to FENO were included [6, 34]. There was no heterogeneity; the result from fixed-effect model was similar to that from random-effects model. These two studies yielded a protective pooled odds risk of 2.16 with 95% CI: 1.05 to 4.43.

### 5.7. Atopic Dermatitis

Two studies focused on atopic dermatitis [5, 34]. There was no heterogeneity, so fixed-effect pooled model was used. These two studies yielded a protective pooled odds risk of 0.60 with 95% CI: 0.42 to 0.85.

### 5.8. Others

The results showed that age at onset, asthma duration, sputum eosinophils, blood eosinophils, and atopy were not important discriminators for airway remodeling in cohort studies.

Five studies found that OR age at onset is another risk factor in IAO asthma patients with OR of 1.04 (95% CI: 1.01–1.06) by the fixed-effect model [4, 5, 8, 36, 40], but significant heterogeneity (*I* ^2^ = 92.4%) was observed among studies and the random-effects model showed different result (OR = 1.02, 95% CI: 0.88–1.19). The amount of heterogeneity did not decrease when the sensitivity analysis was performed (data not shown). We also did not find any evidence of publication bias (*P* value of Egger's symmetry test: 1.00).

Compared with RAO asthma patients, we found longer disease duration (SMD = 2.84, 95% CI: 1.22–4.45), lower sputum eosinophils (SMD = −0.99, 95% CI: 1.20–0.79), and lower body mass index (BMI) (SMD = −1.155, 95% CI: −1.348–0.962) in IAO asthma patients (Table 2).

## 6. Discussion

To our knowledge, this is the first meta-analysis study for the prevalence and the risk factors of IAO. Our findings support the high prevalence of IAO in severe asthma. IAO is highly associated with smoking and male asthma patients have two-fold greater risk of IAO. Furthermore, the asthma patients with higher FENO and aspirin sensitivity also have two-fold greater risk of IAO. However, atopic dermatitis and rhinitis and atopy were significantly related to the decreasing IAO in severe asthma.

The concept of IAO implies that some changes in the structure and function of the airway occur and reduce expiratory airflow, and these changes cannot be restored to the prior state by endogenous mechanism or any treatment. Some indirect evidence demonstrated that progressive airflow obstruction may cause IAO because severe or progressive airflow obstruction occurs in some IAO patients in spite of ongoing anti-inflammatory (glucocorticoid) or bronchodilator treatment. The studies on the pathogenesis of airway remodeling in asthma [43], such as airway wall thickness, allergic airway inflammation, epithelial-driven models of airway remodeling, bronchial neovascularization, and physiological consequences of airway remodeling, support explaining the reasons of IAO.

Some studies have explored the risk factors for the development of IAO in asthma. The reduction in lung function in children and adults may contribute to the development of IAO. Cigarette smoking is related to the development of IAO asthma and has been associated with a rapid decline in lung function in asthma [2, 44]. It might be related to smoking-induced changes in airway inflammation, with a predominance of neutrophils instead of eosinophils [45].

Jiang et al. [46] suggested that atopy in itself is not related to asthma. However, asthma and atopy usually occur in parallel. Since atopic asthma is usually mild and occurs early, it is more commonly accompanied by other allergic diseases such as allergic rhinitis [47]. A reduced prevalence of rhinosinusitis in patients with more severe asthma may be due to the spontaneous regression of rhinosinusitis during the course of asthma [48]. Mascia et al. [11] showed that adults with aspirin-sensitive asthma have more severe disease and airway obstruction than aspirin-insensitive asthma patients. It may be possible that aspirin-sensitive asthma patients have longer disease duration. Chronic inflammation of the airways may also contribute to IAO development, so it may explain why FENO is the major factor of IAO.

Two major mechanisms for airway remodeling including reticular basement membrane (RBM) and subepithelial basement membrane thickening have been reported [49, 50]. Another common finding in remodeled airways is the presence of increased airway smooth muscle mass [51]. Findings of increased RBM thickness and airway smooth muscle area on biopsies of severe asthma patients have intrigued the interest in the use of noninvasive imaging techniques, such as computed tomography (CT) [52], to assess the changes in the bronchial wall thickness and structure. The third dogma is that the structure changes that cause the functional changes in the airway wall are primarily responsible for IAO. In the future, we still use the risk factors to explore and identify the mechanisms of remodeling.

The limitations of this meta-analysis should also be addressed. First, some studies are excluded due to the absence of original dada, which may lead to selection bias. Thus, these studies are usually excluded. Second, all eligible studies are published in English from selected databases. It is possible that some relevant studies published in other languages are missed. Third, most studies are from Asians and Caucasians; thus our study may be applicable to non-Africans only. Fourth, asthma is a heterogeneous disease, and the therapy may affect risk factors. Fifth, we should also analyze the possibility of publication bias. Publication bias can result in the disappearance of some studies with negative results.

In conclusion, this study is the first to report risk factors that may be related to IAO in asthma patients. We found that smoking, male gender, FENO, and the absence of rhinitis, atopy, and atopic dermatitis were more likely to lead to persistent IAO. In addition, our findings further demonstrated that smoking is an important cause of irreversible airway damage. Further we need more studies to clarify the underlying risk factors of the development of IAO in subjects with asthma. The search for known and novel therapies that can directly target individual components of the remodeling process and hopefully lead to an improvement in the treatment of airflow limitation and the prognosis in severe asthma will be made.

## Figures and Tables

**Table 1:** Included studies.

Author	Year	Study design	Country	Population number	Defining IAO	Follow-up time	Increase risk factor	Decrease risk factor
Martinez et al. [41]	1995	Cohort study	Tucson	826	Persistent wheezing	6 yrs	Maternal asthma; maternal smoking; rhinitis apart from colds; Hispanic ethnic background	Rhinosinusitis

Ulrik and Backer [3]	1999	Cohort study	Denmark	31	FEV1 < 80% predicted and change in FEV1 after 5 mg salbutamol < 9% pred	10 yrs	Long-term treatment with oral corticosteroids	—

Ten Brinke et al. [6]	2001	Cohort study	Netherlands	132	Postbronchodilator FEV1 or FEV1/FVC < 75% predicted, using inhaled corticosteroids (1,600 g/d) and/or daily oral prednisone and long-acting bronchodilators for 1 yr	>1 year	Sputum eosinophils ≥ 2%; PC20 histamine 1.0 mg/mL; adult onset of asthma	—

Vonk et al. [8]	2003	Case control	Netherlands	228	FEV1 < 80% predicted and reversibility < 9% predicted	26 years	—	Use of steroids; ln (slope BHR)

Sears et al. [33]	2003	Cohort study	New Zealand	613	FEV1 of less than 75% of the FVC at 9 and 11 years of age and of less than 70% of the FVC at older ages, but spirometry was repeated 10 minutes after they had inhaled nebulized albuterol (5 mg per milliliter) for 2 minutes	26 years	Sex; PC20; positive skin test and age at onset of wheezing	Father smoked when study member was a child

Bumbacea et al. [32]	2004	Case control	UK	66	FEV1 < 50% predicted; these were postbronchodilator FEV1 and had not varied by > 10% when repeated within 3–6 months.	3–6 months	Bronchial thickening and bronchial dilatation; peripheral blood eosinophil	—

Covar et al. [40]	2004	Cohort study	Colorado	990	At least 1% per year loss in postbronchodilator FEV1% predicted. Participants who had a significant reduction in postbronchodilator FEV1% predicted (SRP)	2 yrs	—	Male sex; age

Mascia et al. [11]	2005	Case control (TENOR)	USA	4756	2 or more oral corticosteroid bursts during the 12 months before enrollment, current use of 3 or more medications or chronic daily high doses of inhaled corticosteroids, or use of 5 mg or more of oral prednisone per day.	3-year, multicenter, observational study	Aspirin sensitivity	—

Limb et al. [35]	2005	Cohort study	USA	121	Postbronchodilator FEV1, forced vital capacity, or FEV1/FVC% less than or equal to the 5th percentile or 2 or more indices less than or equal to the 10th percentile (National Health and Nutrition Examination Survey III normative data)	10 yrs	Prematurity	—

Lee et al. [5]	2007	Case control (TENOR study)	USA	1017	Postbronchodilator FEV1/FVC ratio < 70% at two annual consecutive visits	2 yrs	Older age; Black patients; male; smoking; aspirin sensitivity	Hispanic ethnicity; college education or advanced degree; family history of atopic dermatitis; dust sensitivity

Jang et al. [36]	2007	Case control	Korea	582	FEV1/FVC and a predicted FEV1 of < 75%	1 yr	Age and asthma duration	BMI

Liebhart et al. [37]	2008	Case control	Poland	110	The predicted value of FEV1% predicted after bronchodilator administration < 80%	?	Male; disease duration; smoking	—

Kaminska et al. [32]	2009	Case control	Canada	34	Prebronchodilator FEV1 < 70% of predicted value and FEV1/FVC ratio < 80% of predicted value at each visit	At least 12 months	—	—

Contoli et al. [31]	2010	Case control	Italy	31	Postbronchodilator FEV1/FVC < 70%	5 yrs	—	—

Jang et al. [38]	2010	Cohort study	Korea	674	FEV1/FVC and a predicted FEV1 of <75% following bronchodilator	1 yr	Asthmatics without rhinitis	—

Lee et al. [4]	2011	Case control	Korea	235	FEV1/FVC ratio < 70% on all of three pulmonary function tests despite use of high-dose inhaled corticosteroids (ICS) and long-acting b2-agonists (LABA)	3 months	Smoking more than 5 pack-years; asthma more than 15 years	Rhinosinusitis

Perret et al. [39]	2013	Cohort study	USA	8,583	Postbronchodilator FEV1/FVC less than the lower limit of normal, regardless of postbronchodilator reversibility	44 yrs	Smoking; atopy	—

Gupta et al. [30]	2007	Case control	USA	73	Daily high-dose administration of ICS ≥ 800 mg budesonide or ≥ 400 mg fluticasone/momethasone per day in combination with LABA and/or LTRA	1 year	—	—

FEV1: forced expiratory volume in 1 second; FEV1/FVC: forced expiratory volume in 1 second/forced vital capacity; BMI: body mass index; yr: year.

**Table 2:** Pooled odds risks (OR) and 95% confidence intervals (CIs) of irreversible airway obstruction asthma and risk factor in cohort or case control study.

Type	Number of studies	OR (95% CI) Fixed effects	OR (95% CI) Random effects	*I* ^2^
Age	5 (cohort)	**1.037 (1.012, 1.062)**	1.023 (0.878, 1.192)	92.4% (R)
Male	10 (case control)	**2.222 (1.821, 2.731)**		53.3% (F)
Age at onset	4 (cohort)	0.971 (0.937, 1.006)	1.166 (0.646, 2.103)	47.2% (R)
Disease duration	5 (cohort)	1.062 (1.038, 1.086)	1.158 (1.026, 1.308)	91.6% (R)
Rhinitis	3 (case control)	**0.31 (0.24, 0.40)**		0.0% (F)
Atopic dermatitis	2 (cohort)	**0.599 (0.424, 0.848)**		2.1% (F)
Aspirin sensitivity	2 (cohort)	2.053 (1.417, 2.689)	1.826 (0.263, 12.693)	92.4% (R)
Smoking	**9 (case control)**	**1.79 (1.46, 2.19)**	—	**38.2% (F)**
Sputum eosinophils	2 (cohort)	0.979 (0.937, 1.022)	2.514 (0.334, 18.893)	91.6% (R)
Blood eosinophils	2 (cohort)	2.031 (0.958, 4.304)		32.4% (F)
FENO	2 (cohort)	**2.156 (1.050, 4.425)**		0.0% (F)
Atopy	8 (case control)	0.584 (0.466, 0.732)	—	22.2 (F)

R: randomised effects; F: fixed effects.
